# Antiproliferative and Antimigration Activities of Fluoro-Neplanocin A via Inhibition of Histone H3 Methylation in Triple-Negative Breast Cancer

**DOI:** 10.3390/biom10040530

**Published:** 2020-03-31

**Authors:** Woong Sub Byun, Won Kyung Kim, Ji-seong Yoon, Dnyandev B. Jarhad, Lak Shin Jeong, Sang Kook Lee

**Affiliations:** 1 College of Pharmacy, Natural Products Research Institute, Seoul National University, Seoul 08826, Korea; sky_magic@naver.com (W.S.B.); dnjsrud6764@naver.com (W.K.K.); 2 College of Pharmacy, Research Institute of Pharmaceutical Sciences, Seoul National University, Seoul 08826, Korea; mrx666knight@hotmail.com (J.-s.Y.); djarhad12@gmail.com (D.B.J.); lakjeong@snu.ac.kr (L.S.J.)

**Keywords:** triple-negative breast cancer, DOT1L, histone H3 lysine methylation, H3K79me2, fluoro-neplanocin A, metastasis

## Abstract

Triple-negative breast cancer (TNBC) is among the most aggressive and potentially metastatic malignancies. Most affected patients have poor clinical outcomes due to the lack of specific molecular targets on tumor cells. The upregulated expression of disruptor of telomeric silencing 1-like (DOT1L), a histone methyltransferase specific for the histone H3 lysine 79 residue (H3K79), is strongly correlated with TNBC cell aggressiveness. Therefore, DOT1L is considered a potential molecular target in TNBC. Fluoro-neplanocin A (F-NepA), an inhibitor of *S*-adenosylhomocysteine hydrolase, exhibited potent antiproliferative activity against various types of cancer cells, including breast cancers. However, the molecular mechanism underlying the anticancer activity of F-NepA in TNBC cells remains to be elucidated. We determined that F-NepA exhibited a higher growth-inhibitory activity against TNBC cells relative to non-TNBC breast cancer and normal breast epithelial cells. Moreover, F-NepA effectively downregulated the level of H3K79me2 in MDA-MB-231 TNBC cells by inhibiting DOT1L activity. F-NepA also significantly inhibited TNBC cell migration and invasion. These activities of F-NepA might be associated with the upregulation of E-cadherin and downregulation of N-cadherin and Vimentin in TNBC cells. Taken together, these data highlight F-NepA as a strong potential candidate for the targeted treatment of high-DOT1L-expressing TNBC.

## 1. Introduction

Breast cancer (BC) is the most commonly occurring cancer and second leading cause of cancer-related death in women worldwide [1,2]. Chemotherapy, radiation therapy, and surgical options such as mastectomy or lumpectomy have been identified as the most effective and common strategies for the treatment of early-stage BC. However, approximately 40% of patients with BC face a risk of cancer recurrence within a few years of treatment [3,4,5]. Recurrent BCs are not only highly metastatic but also tend to acquire resistance to previously used conventional therapies, including hormone therapy, chemotherapy, or targeted drugs. Consequently, 90% of deaths attributed to BC are associated with recurrent and/or metastatic disease [6,7].

It is reported that approximately 20% of BCs do not express hormonal receptors (estrogen receptor and progesterone receptor) and the human epidermal growth factor receptor 2 (HER2). Therefore, these BCs are classified as triple-negative BCs (TNBCs) [8,9]. On this line, the TNBCs do not respond to hormonal or targeted therapy and are also considered the most intractable and aggressive form of cancer. Recent reports highlighted the specific overexpression of genes related to the epithelial–mesenchymal transition (EMT), an essential regulator of the process of cancer metastasis, in TNBCs relative to other common types of BC [2,10,11]. Many attempts have been made to develop effective drugs for the treatment of metastatic TNBCs. However, existing therapeutic options such as docetaxel-based regimens are highly toxic to normal cells and induce significant adverse effects. Therefore, novel strategies to treat patients with aggressive and metastatic TNBC are strongly needed.

Epigenetic modulations, including DNA methylation and histone modification, act as essential regulators of many biological processes considered fundamental to the maintenance of gene expression patterns and development [12,13]. However, the aberrant regulation of epigenetic processes can lead to tumor initiation, malignant cellular transformation, and altered gene functions [12,14,15]. Each epigenetic process is regulated by specific enzymes and their interactions. Disruptor of telomeric silencing 1-like (DOT1L), a histone methyltransferase specific for the lysine 79 residue of histone H3 (H3K79), was recently reported to promote the development of mixed lineage leukemia (MLL)-rearranged leukemia and the proliferation and metastasis of BC [2,16,17,18,19]. Although this discovery has led to a clinical trial of the DOT1L inhibitor EPZ-5676 for the treatment of leukemias harboring a rearrangement of the MLL gene, existing DOT1L inhibitors did not sufficiently suppress breast tumor growth and metastasis in an earlier study [20]. These findings prompted us to develop a novel DOT1L inhibitor for BC, especially for TNBCs which are refractory to existing targeted therapeutic agents.

Adenosine, a naturally occurring nucleoside, functions as an important intermediary metabolite and signaling molecule in humans. Consequently, adenosine signaling has been used as a molecular target in various diseases since the 1940s. Still, it is difficult to determine the systemic effects of direct adenosine-targeted therapies, as this molecule is metabolized easily and decomposes rapidly into inosine and AMP (Adenosine monophosphate). Therefore, strategies for targeting adenosine signaling mainly aim to block or modulate adenosine receptors using chemically synthesized adenosine analogs [21,22,23]. A large body of evidence indicates an association of dysregulated adenosine signaling with cancer cell differentiation and tumor progression, and nucleoside-type analogs now comprise a main class of small molecule anticancer agents [24,25,26,27]. Particularly, neplanocin A (NepA), a naturally occurring cyclopentenyl analog of adenosine with notable antiviral activity, significantly inhibited the proliferation of cancer cells by inhibiting *S*-adenosylhomocysteine hydrolase (SAH) [28,29,30], an enzyme that catalyzes the hydrolysis of *S*-adenosylhomocysteine into _L_-homocysteine and adenosine. The inhibition of SAH leads to the intracellular accumulation of *S*-adenosylhomocysteine and further inhibits the *S*-adenosylmethionine-dependent process of transmethylation required for viral replication [31,32]. However, the presence of an acidic hydrogen at the 4′-position of NepA results in an incomplete and reversible inhibition of SAH. Consequently, we previously designed and synthesized halo-NepA analogs to perpetuate the inhibition of SAH. Unlike NepA, halo-NepA analogs yielded a novel and irreversible form of inhibition against SAH. Particularly, fluoro-NepA (F-NepA) exhibited more potent inhibitory activity against SAH than the parent compound [33,34].

DOT1L methylates H3K79 via the methyl group of *S*-adenosylmethionine (SAM) [35,36]. However, the potential growth-inhibiting effects of SAH inhibition on BC cells and the underlying molecular mechanisms remain to be elucidated. Given the remarkable inhibitory activity of F-NepA on SAH, we considered that this agent could potentially target histone methylation in BC cells and might thus yield better treatment outcomes for treatment-refractory cancers such as TNBC. Herein, we report for the first time the antiproliferative and antimigration activities of F-NepA in human TNBC cells. Notably, we determined that the growth-inhibitory activity of F-NepA might be associated partly with the suppression of DOT1L expression in TNBC cells. Moreover, F-NepA effectively inhibited the migration and invasion of TNBC cells by modulating EMT processes. These findings suggest that F-NepA might be applicable as a novel and strong candidate chemotherapeutic agent for the treatment of human TNBC.

## 2. Materials and Methods

### 2.1. Cell Culture and Chemicals

Human breast epithelial cell (MCF10A) and BC cell lines (HCC38, HCC1937, MDA-MB-231, MCF-7, T-47D) were obtained from the American Type Culture Collection (Manassas, VA, USA). The cells were cultured in medium (Dulbecco’s Modified Eagle’s Medium/Nutrient Mixture F-12 for MCF10A cells; Roswell Park Memorial Institute 1640 medium for HCC38, HCC1937, and T-47D cells; Dulbecco’s Modified Eagle’s Medium for MDA-MB-231 and MCF-7 cells) supplemented with penicillin-streptomycin (10,000 units/mL sodium penicillin G and 10,000 μg/mL streptomycin) and 10% fetal bovine serum (FBS) in an incubator containing 5% CO_2_ at 37 °C. All reagents used for cell culture were purchased from Gibco^®^ Invitrogen Corp. (Grand Island, NY, USA). Etoposide and sulforhodamine B were purchased from Sigma-Aldrich (St. Louis, MO, USA). Recombinant human TGF-β1 was purchased from PeproTech (Rocky Hill, NJ, USA). Analogs of NepA were provided by coauthor Dr. Lak Shin Jeong [30,37]. The stock solutions were dissolved in 100% DMSO.

### 2.2. Western Blotting Analysis

Total cell lysates were prepared in 2× sample loading buffer [250 mM Tris-HCl (pH 6.8), 10% glycerol, 4% sodium dodecyl sulfate (SDS), 2% β-mercaptoethanol, 0.006% bromophenol blue, 5 mM sodium orthovanadate, and 50 mM sodium fluoride; Bio-Rad, Hercules, CA, USA]. The protein concentrations of samples were quantified using the bicinchoninic acid (BCA) method [38] and a BCA Protein Assay Kit (Thermo Fisher Scientific, Waltham, MA, USA). Equal amounts of protein (5–20 μg) were separated by 6–13% SDS-polyacrylamide gel electrophoresis (PAGE) and transferred to polyvinylidene fluoride membranes (Millipore, Bedford, MA, USA). The membranes were blocked with 5% bovine serum albumin (Sigma-Aldrich) and then probed with anti-DOT1L, anti-H3K79me2, anti-β-Actin, anti-histone H3, anti-H3K4me2, anti-H3K9me2, anti-H3K27me2, anti-H3K36me2, and anti-Vimentin antibodies purchased from Cell Signaling Technology (Beverly, MA, USA) or with anti-E-cadherin and anti-N-cadherin antibodies purchased from BD Biosciences (San Jose, CA, USA). The blots were detected using a WEST-Queen detection system (iNtRON Biotechnology, Seongnam, Republic of Korea).

### 2.3. Cell Proliferation Assay (SRB assay)

Cell proliferation was measured using a sulforhodamine B (SRB) assay [39]. Briefly, cells were seeded in 96-well plates and incubated for 30 min (for day 0 controls) or treated with test compounds for the indicated times. After incubation, the cells were fixed, dried, and stained with 0.4% SRB in 1% acetic acid solution. Unbound dye was removed by washing with 1% acetic acid, after which the stained cells were dissolved in 10 mM Tris (pH 10.0). The absorbance of the cell solution was measured at 515 nm, and this value was used to determine cell proliferation. IC_50_ values were calculated by a nonlinear regression analysis with TableCurve 2D v5.01 software (Systant Software Inc., Richmond, CA, USA). All reagents used in the SRB assay were purchased from Sigma-Aldrich.

### 2.4. DOT1L Enzyme Activity Assay

DOT1L enzyme activity was measured using S-adenosyl methionine (SAM) as the methyl group donor and synthesized DOT1L as the substrate (Cat. No. 52202; BPS Bioscience, San Diego, CA, USA) according to the manufacturer’s instructions.

### 2.5. Cell Viability (MTT Assay)

Cell viability was measured using an MTT assay. MDA-MB-231 cells were seeded in 12-well plates and incubated overnight. Next, the cells were treated with the indicated concentrations of F-NepA (0.2–3.2 μM) in growth media for the indicated times. Subsequently, the cells were gently washed twice with growth medium and incubated with 0.5 mg/mL MTT (Sigma-Aldrich) at 37 °C for 4 h. The formazan crystals formed by active mitochondria were dissolved in DMSO, and the absorbance in each well was measured at 570 nm and used to determine cell viability. The IC_50_ values were calculated by a nonlinear regression analysis using TableCurve 2D v5.01 software (Systant Software Inc.). All reagents used in the MTT assay were purchased from Sigma-Aldrich.

### 2.6. Wound Healing Assay

MDA-MB-231 and HCC1937 human TNBC cells were grown to 90% confluence in a six-well plate. Subsequently, each cell monolayer was artificially wounded using a Scratcher (SPL Life Sciences, Pocheon, Republic of Korea), and the detached cells were removed by washing with phosphate-buffered saline (PBS, Invitrogen Corp.). The wounded cultures were then incubated with medium containing 1% FBS and various concentrations of F-NepA for 24 h. The wounds were photographed at 0 and 24 h under an inverted microscope (Olympus, Tokyo, Japan). The wound areas were quantified using ImageJ software (National Institutes of Health, Bethesda, Maryland, USA) and presented as the percent wound healing (%) relative to the wound area at 0 h [40].

### 2.7. Transwell Cell Invasion Assay

Twenty-four-well Transwell membrane inserts (diameter: 6.5 mm, pore size: 8 μm; Corning, Tewksbury, MA, USA) were each coated with 10 μl of type I collagen (0.5 mg/mL, BD Biosciences, San Diego, CA, USA) and 20 μl of a 1:20 mixture of Matrigel (BD Biosciences) in PBS. After treatment with the indicated compounds for 24 h, MDA-MB-231 and HCC1937 human TNBC cells were harvested, resuspended in serum-free medium, and plated (2 × 10^5^ cells/chamber) in the upper chambers of the Matrigel-coated Transwell inserts. Medium containing 30% FBS was used as a chemoattractant in the lower chambers. After a 24 h incubation, the cells that had migrated to the outer surfaces of the lower chambers were fixed and stained using the Diff-Quik Staining Kit (Sysmex, Kobe, Japan) and imaged using a Vectra 3.0 Automated Quantitative Pathology Imaging System (Perkin Elmer, Waltham, MA, USA). Representative images from three separate experiments are shown, and the numbers of invaded cells were counted in five randomly selected microscopic fields (200× magnification) [41].

### 2.8. RNA Isolation and Real-Time Polymerase Chain Reaction (Real-Time PCR) Analysis

Total RNA was extracted from the cells using NucleoZOL reagent (Macherey-Nagel, Bethlehem, PA, USA). One microgram of total RNA per sample was reverse transcribed using a Reverse Transcription System (Cat. No. A3500; Promega, Madison, WI, USA) according to the manufacturer’s instructions. Real-time PCR was conducted using iQ SYBR Green Supermix (Bio-Rad, Hercules, CA, USA) according to the manufacturer’s instructions. The threshold cycle (C_T_) was determined using Bio-Rad CFX manager 3.1 software. After normalizing the expression data to the level of the housekeeping gene *ATCB* (β-Actin mRNA), the comparative C_T_ method was used to calculate the relative differences in mRNA expression between compound-treated cells and untreated controls [42]. The following primers were used for real-time PCR: *CDH1,* 5′-GTT ATT CCT CTC CCA TCA GCT G-3′ and 5′-CTT GGC TGA GAG GAT GGT GTA A-3′; *CDH2*, 5′-AGC CAA CCT TAA CTG AGG AGT-3′ and 5′-GGC AAG TTG ATT GGA GGG ATG-3′; *VIM*, 5′-AGA TGG CCC TTG ACA TTG AG-3′ and 5′-TGG AAG AGG CAG AGA AAT CC; *ATCB,* 5′-AGC ACA ATG AAG ATC AAG AT-3′ and 5′-TGT AAC GCA ACT AAG TCA TA-3′.

### 2.9. Statistical Analysis

The data are presented as mean values ± standard deviations (SDs) for the indicated numbers of independently performed experiments. All data are representative of the results of at least three independent experiments. Statistical significance was analyzed using Student’s t-test or a one-way analysis of variance coupled with Dunnett’s *t*-test. Differences were considered statistically significant at ^*^p < 0.05, ^**^p < 0.01, ^***^p < 0.001.

## 3. Results

### 3.1. Expression Levels of DOT1L and Antiproliferative Activities of NepA Analogs in Human BC Cells

**Figure 1:**
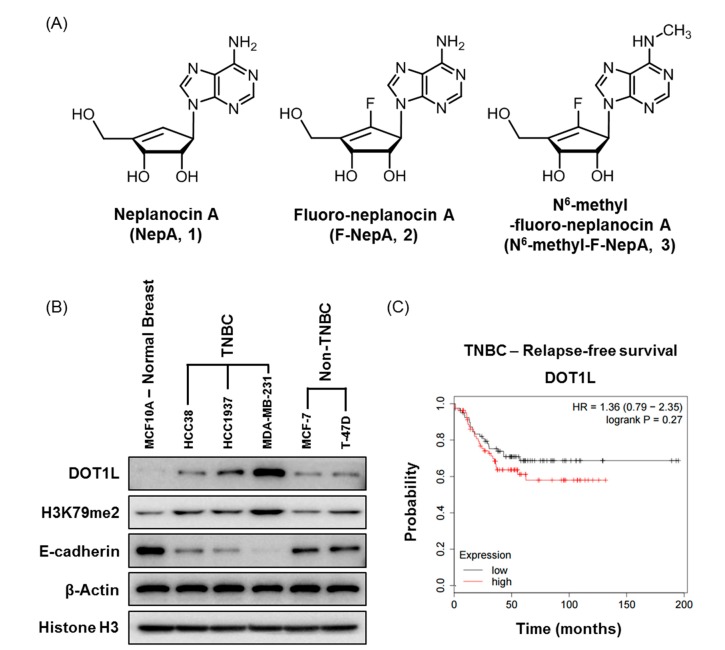
DOT1L expression levels in BCs and correlation between DOT1L expression and relapse-free survival in patients with TNBC. (**A**) Structures of NepA analogs: NepA, **1**; F-NepA, **2**; N^6^-methyl-F-NepA, **3**. (**B**) DOT1L, H3K79me2, and E-cadherin levels in various BC cell lines were detected by Western blotting. β-Actin and Histone H3 were used as an internal control. (**C**) The Kaplan–Meier survival curve represents the relapse-free survival durations of patients with TNBC according to the DOT1L (Affy ID: 226201_at) expression level.

**Figure 2:**
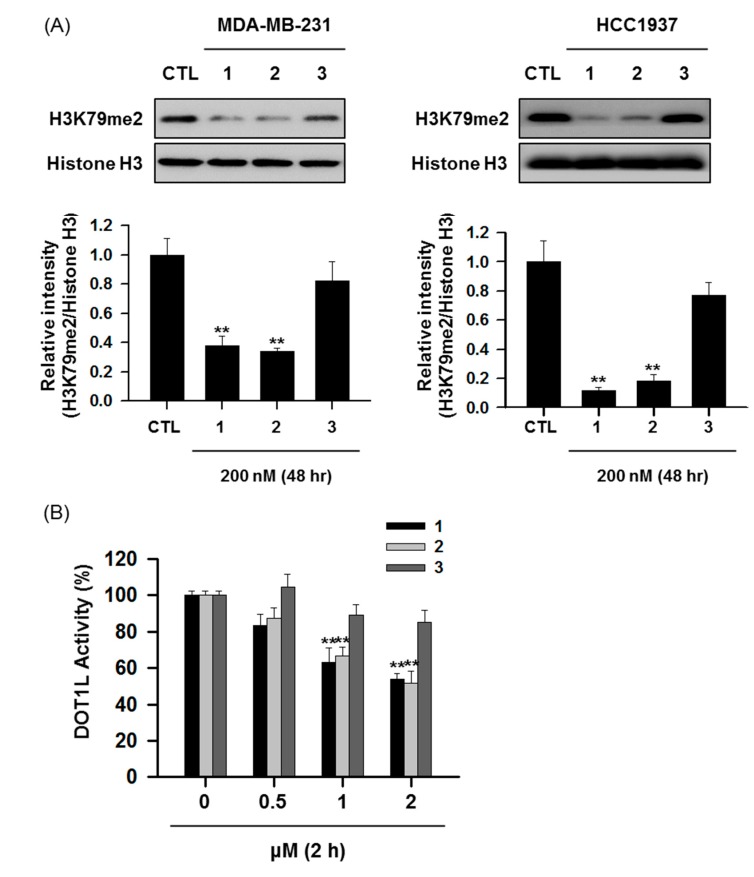
Suppressive effects of NepA analogs on H3K79me2 via the inhibition of DOT1L. (**A**) MDA-MB-231 and HCC1937 cells were treated with NepA analogs (200 nM) for 48 h, and H3K79me2 levels were determined by Western blotting. Histone H3 was used as an internal control. The relative intensities of the indicated protein levels were analyzed semi-quantitatively using NIH ImageJ software. (**B**) DOT1L (500 ng/well) enzyme activity levels were analyzed after incubation with NepA analogs (0.5–2 μM) for 2 h. All data are expressed as mean values ± SD (n = 3) and are representative of three separate experiments. **p < 0.01 indicates significant differences relative to the vehicle-treated control group.

**Figure 3:**
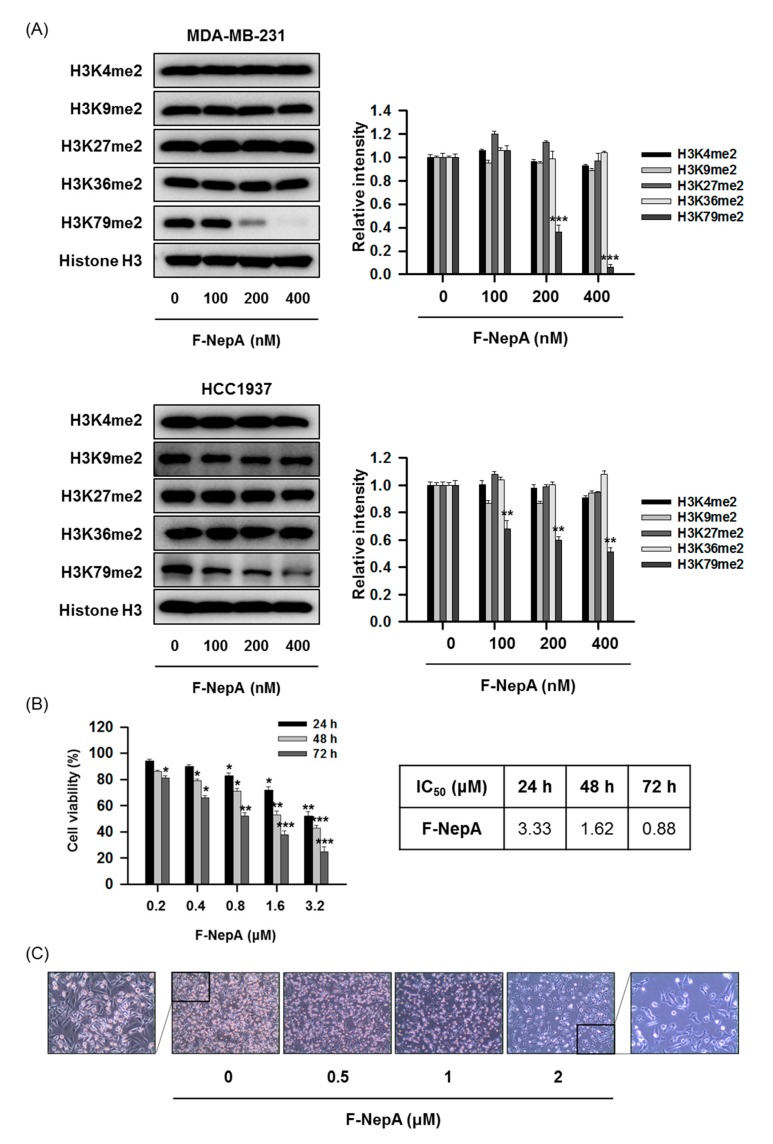
Effects of F-NepA on the methylation of various H3 lysine residues and viability in human TNBC cells. (**A**) MDA-MB-231 and HCC1937 cells were treated with the indicated concentrations of F-NepA for 48 h, and the methylation levels of various H3 lysine residues were determined by Western blotting. Histone H3 was used as an internal control. The relative intensities of the indicated proteins were analyzed semi-quantitatively using NIH ImageJ software. (**B**) MDA-MB-231 cells were incubated with the indicated concentrations of F-NepA for 24–72 h, after which cell viability was measured using an MTT assay. Data are expressed as means ± SD from three independent experiments. The IC_50_ values were calculated via a nonlinear regression analysis using TableCurve 2D v5.01 software. (**C**) Morphological changes in MDA-MB-231 cells induced by F-NepA (0–2 μM) were viewed under an inverted phase-contrast microscope (100× magnification). All data are expressed as mean values ± SD (n = 3) and are representative of three separate experiments. *p < 0.05, **p < 0.01, and ***p < 0.001 indicate significant differences relative to the vehicle-treated control group.

We previously designed and synthesized analogs of NepA (Figure 1A, **1**) and reported the antiproliferative activities of these molecules against a panel of human cancer cell lines, including A549 (lung cancer cells), HCT-116 (colorectal cancer cells), SNU-638 (stomach cancer cells), MDA-MB-231 (BC cells), SK-HEP-1 (liver cancer cells), and PC-3 (prostate cancer cells). Of these analogs, F-NepA (Figure 1A, **2**) and N^6^-methyl-F-NepA (Figure 1A, **3**) exhibited potent growth-inhibitory activity against human cancer cells [37]. Our findings suggested that the inhibitory effect of NepA on the production of SAM via the inhibition of SAH might also affect the histone methylation status of human cancer cells. In BC, the upregulated methylation of H3K79 (H3K79me) is known to be correlated strongly with a poor clinical outcome [2]. Therefore, we focused on DOT1L expression and DOT1L-mediated methylation levels in BC cells. As shown in Figure 1B, the TNBC cell lines HCC38, HCC1937, and MDA-MB-231 exhibited higher DOT1L and H3K79me2 expression levels relative to those in normal breast epithelial (MCF10A) and non-TNBC cell lines (MCF-7, T-47D). In comparison, the expression level of E-cadherin, an epithelial marker, was found to be significantly low in TNBC cell lines. We further analyzed the clinical significance of DOT1L expression in TNBC patients with respect to relapse-free survival (RFS) according to the Kaplan−Meier method. We applied the auto-select best cutoff method to classify patients with BC. Patients were further classified according to their levels of DOT1L expression and probability of RFS. Among patients with TNBC, those with high DOT1L expression had a lower probability of RFS than did those with low DOT1L expression (Figure 1C). We also found that NepA and its analogs exhibited more potent antiproliferative activity against TNBC cells relative to non-TNBC cells. In particular, F-NepA (**2**) showed a similar antiproliferative activity with NepA (**1**) against human BC cells, but F-NepA was less cytotoxic compared to NepA against normal breast epithelial cells (MCF10A). These findings indicate that the introduction of fluorine may reduce the cytotoxicity associated with NepA in normal cells (Table 1). In addition, the MDA-MB-231 and HCC1937 cells exhibited the relatively higher DOT1L expression levels and downregulated E-cadherin level among the tested human breast cancer cell lines. Therefore, these two cell lines were employed in subsequent experiments as representative of TNBC cells.

### 3.2. Effects of NepA Analogs on DOT1L Activity and H3K79me2 Expression

To further determine whether NepA analogs downregulated the methylation status of H3K79, we treated TNBC cells (MDA-MB-231 and HCC1937 cells) with NepA analogs for 48 h and analyzed the H3K79me2 levels by Western blotting. As expected, NepA (**1**) and F-NepA (**2**) exhibited remarkable abilities to suppress H3K79 dimethylation at the test concentration of 200 nM. In contrast, the N^6^-methyl derivative of F-NepA (**3**) exhibited negligible inhibitory activity against H3K79me2 (Figure 2A). In addition, a DOT1L enzymatic activity assay confirmed that NepA and F-NepA effectively inhibited DOT1L activity under cell-free conditions (Figure 2B), suggesting that the suppressive effects of NepA analogs on the H3K79me2 levels in TNBC cells were associated with the inhibition of DOT1L enzymatic activity. Furthermore, the introduction of an N^6^-methyl group into F-NepA reduced the cancer cell antiproliferative activity of this analog and the DOT1L-mediated suppression of H3K79me2, indicating that the N^6^-amine moiety is a pivotal structural chemical component in the bioactivity of NepA analogs (Table 1, Figure 2).

### 3.3. F-NepA Selectively Suppresses DOT1L-Mediated H3K79 Methylation in Human TNBC Cells

Despite the potent antiproliferative activity exhibited by NepA against numerous types of human cancer cells, the high toxicity and reversible SAH inhibition associated with this agent has limited its clinical development and use [30,43]. Compared to NepA, F-NepA irreversibly inhibits SAH and thus exhibits great potential as a therapeutic agent that targets TNBCs with upregulated DOT1L expression. The methylation of each H3 residue is governed by a specific enzyme (e.g., SET1A/B for H3K4, G9a for H3K9, or EZH1/2 for H3K27) [44,45]. After confirming the potency of F-NepA against DOT1L, we further evaluated whether F-NepA would affect the methylation of specific H3 lysine residues in human TNBC cells. Although treatment with F-NepA for 48 h effectively suppressed dimethylation at H3K79 in a concentration-dependent manner, no significant effects were observed on other H3 residues (Figure 3A). These findings suggest that F-NepA selectively suppresses DOT1L, as indicated by the downregulation of H3K79me2, without altering the activities of other histone methyltransferases. Moreover, as shown in Figure 3B, F-NepA exhibited cytotoxicity against MDA-MB-231 in a time- and concentration-dependent manner. This analog also evoked significant morphological changes in treated cells, which exhibited either shrunken or pointed shapes relative to the appearances of vehicle-treated control cells when analyzed under an inverted phase-contrast microscope (Figure 3C). These morphological changes might be partly concomitant with mesenchymal–epithelial transition induced by treatment of F-NepA.

### 3.4. F-NepA Modulates EMT-Associated Gene Expressions in Human TNBC Cells

**Figure 4:**
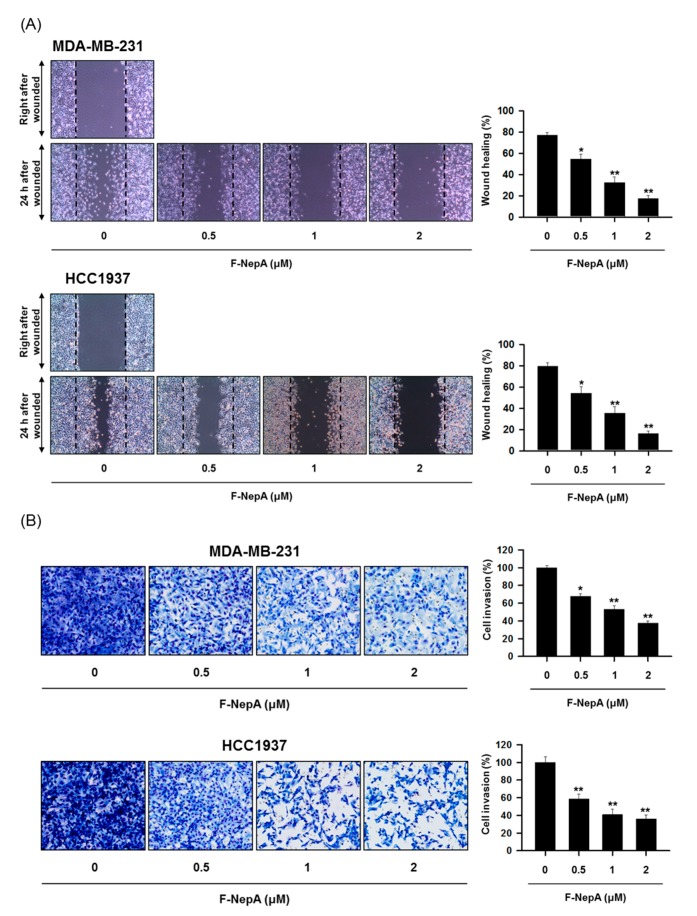
Regulatory effects of F-NepA on the migration and invasion potential of human TNBC cells. (**A**) Monolayers of MDA-MB-231 and HCC1937 cells were scratched mechanically and treated with F-NepA for 24 h. Representative images of wound closure obtained under a light microscope are shown (left). The areas of the wounds were quantified using ImageJ (right). (**B**) MDA-MB-231 and HCC1937 cells were pretreated with F-NepA at the indicated concentrations for 24 h, reseeded into the upper chambers of Transwell inserts, and incubated for 24 h. The cells that invaded the lower chambers were fixed, stained, imaged (left), and counted (right). *p < 0.05 and **p < 0.01 indicate significant differences relative to the vehicle-treated control group.

**Figure 5:**
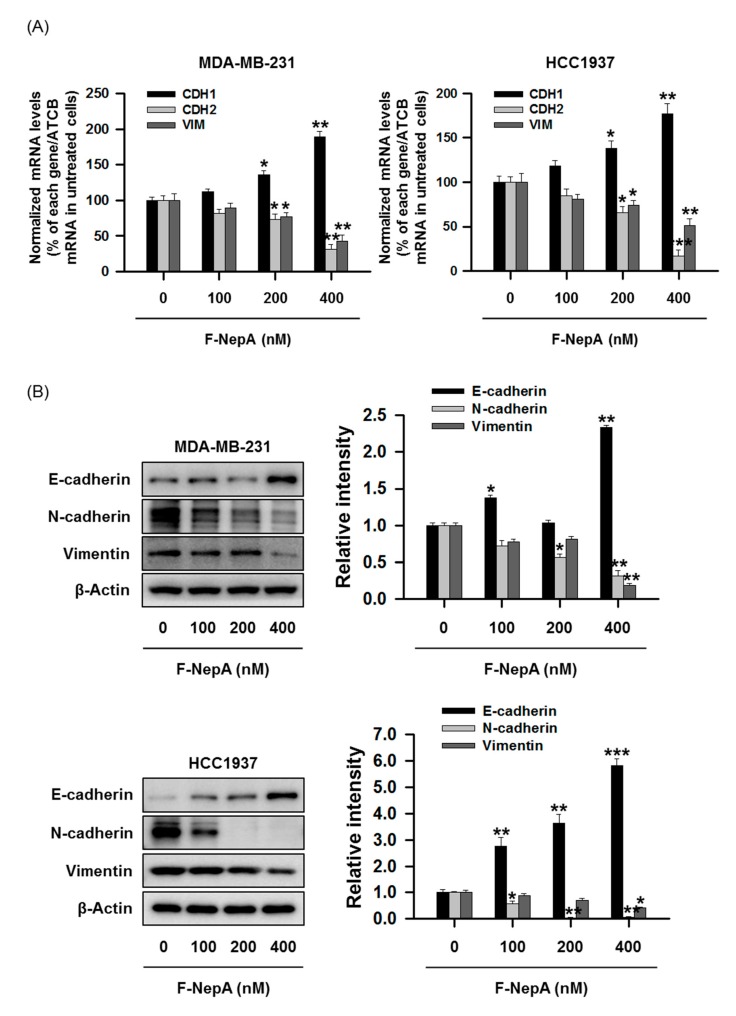
F-NepA regulates EMT gene expression in human TNBC cells. (**A**) MDA-MB-231 and HCC1937 cells were treated with F-NepA at the indicated concentrations for 24 h, and the mRNA levels of *CDH1*, *CDH2*, and *VIM* were determined using real-time PCR. (**B**) The protein levels of E-cadherin, N-cadherin, and Vimentin in MDA-MB-231 and HCC1937 cells treated with F-NepA for 48 h were determined by Western blotting. β-Actin was used as an internal control. The relative intensities of the indicated proteins were analyzed semi-quantitatively using NIH ImageJ software. The data are expressed as the mean values ± SD (n = 3) and are representative of three separate experiments. *p < 0.05, **p < 0.01, and ***p < 0.001 indicate significant differences relative to the vehicle-treated control group.

TNBCs exhibiting aberrant DOT1L expression eventually undergo EMT. This process is strongly associated with cancer cell metastasis to other organs [46], which is a main cause of lethality in all BC cases [47]. Since cancer metastasis is a multistep process including cell migration and invasion, we determined whether F-NepA is able to regulate the metastatic potential of human TNBC cells via the inhibition of these two processes which might be coincident with DOT1L expression. Notably, F-NepA treatment of MDA-MB-231 and HCC1937 cells for 24 h effectively inhibited wound closure in a wound healing assay (cell migration, Figure 4A) and cell invasion in a Transwell-invasion assay (Figure 4B) in a concentration-dependent manner. To further elucidate the molecular mechanism underlying the inhibition of cancer cell migration and invasion by F-NepA, we evaluated the effects of F-NepA on the expression levels of EMT-associated genes using real-time PCR and Western blotting. Treatment with F-NepA for 24 h induced the upregulation of *CDH1*, which encodes the epithelial biomarker E-cadherin, and downregulated the expression of the genes *CDH2* and *Vim*, which respectively encode the typical mesenchymal biomarkers N-cadherin and Vimentin, in human TNBC cells (Figure 5A). In the same cell lines, treatment with F-NepA for 48 h similarly regulated the protein levels of EMT markers, including E-cadherin, N-cadherin, and Vimentin, in a concentration-dependent manner (Figure 5B). These results suggest that the antimigration and anti-invasion activities of F-NepA might be associated with the suppression of the DOT1L-dependent EMT signaling pathway in TNBC cells.

### 3.5. F-NepA Reverses TGF-β Induced EMT Biomarker Expressions

**Figure 6:**
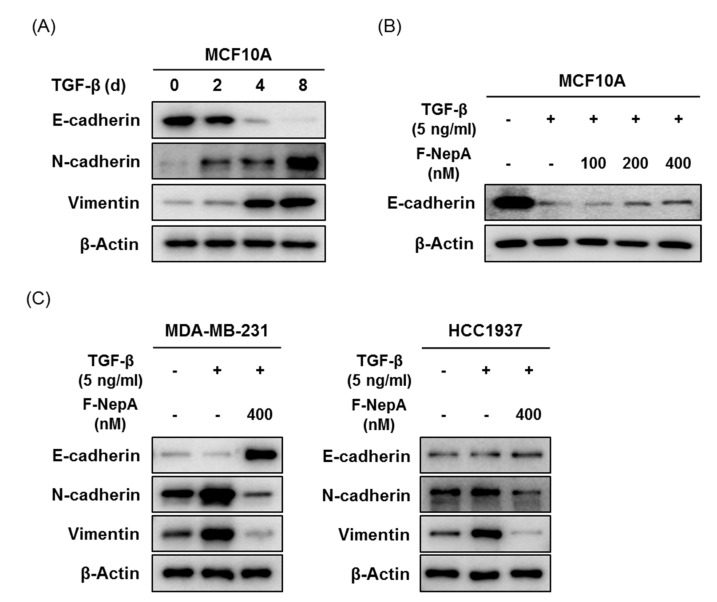
F-NepA reverses TGF-β induced EMT biomarker expressions. (**A**) MCF10A cells were treated with TGF-β1 (5 ng/mL) for indicated time periods, and the protein expression of E-cadherin, N-cadherin, and Vimentin was analyzed by Western blotting. β-Actin was used as an internal control. (**B**) MCF10A cells were treated in the presence or absence of TGF-β1 (5 ng/mL) for 8 days, and then fresh media without TGF-β1 were replaced and treated with indicated concentrations of F-NepA for an additional 2 days. The expression of E-cadherin was analyzed by Western blotting. β-Actin was used as an internal control. (**C**) MDA-MB-231 and HCC1937 cells were treated in the presence or absence of TGF-β1 (5 ng/mL) for 8 days, and then fresh media were replaced without TGF-β1 and treated with indicated concentrations of F-NepA for an additional 2 days. The expression of E-cadherin, N-cadherin, and Vimentin was analyzed by Western blotting. β-Actin was used as an internal control.

EMT is a complex and reversible process that is triggered by signals, such as hypoxic condition, TGF- β, and TNF-α [19]. Since the upregulation of the EMT signaling pathway is known to be a main cause of tumor metastasis, we further designed and performed the experiments for evaluating the effect of F-NepA in TGF-β-induced EMT models. Preferentially, to confirm the effect of TGF-β on induction of EMT signaling molecules, MCF10A, a normal breast epithelial cell line, was treated with TGF-β (5 ng/mL) for indicated times (days). As a result, TGF-β treatment effectively downregulated the expression of an epithelial marker (E-cadherin), and upregulated the expression of mesenchymal markers (N-cadherin and Vimentin) in a time-dependent manner (Figure 6A). The downregulated E-cadherin expression in TGF-β-induced MCF10A cells was slightly recovered by F-NepA treatment for 48 h (Figure 6B). Similarly, F-NepA effectively upregulated E-cadherin expression and downregulated N-cadherin and Vimentin expressions in two TGF-β-induced highly metastatic human TNBC cells (Figure 6C). Taken together, these data suggest that the TGF-β-induced EMT process could be effectively reversed by F-NepA treatment in breast epithelial cells as well as in highly metastatic TNBC cells.

## 4. Discussion

Nucleosides, which are endogenous to all cells of the body, are essential to the maintenance and regulation of numerous biological functions, including signaling pathways, energy metabolism, and heredity control [48,49]. Since the first descriptions of the chemical synthesis of adenosine and guanosine in the 1940s, modified nucleosides have become valuable therapeutic agents in an enormous number of situations because of their relative safety and significant activity levels [25,50]. Nucleoside analogs comprise a main class of small molecule-based antiviral, antitumor, and antibacterial agents [28,51,52]. Previously, we reported the synthesis and biological activity of halo-analogs of NepA, among which we identified F-NepA as a significantly more potent inhibitor of SAH relative to the parent compound, whereas chloro- and bromo-substituted analogs were relatively less active [33]. In another recent report [37], we described the profound antiproliferative activity of F-NepA against a panel of cancer cells. Therefore, in this study, we aimed to determine whether a decrease in SAM via the F-NepA-mediated inhibition would also affect the methylation status of H3 in human TNBC cells.

The regulation of aberrantly activated H3 methyltransferases is considered an attractive anticancer strategy [53,54]. In this regard, DOT1L plays a major role in regulating gene expression and chromosome structure during development and gene transcription by targeting H3K79 [55]. However, recent findings suggest that the dysregulation of DOT1L expression and activity is closely associated with aggressive behavior and metastatic potential in human TNBC cells [19]. Accordingly, we analyzed the relationships between the clinical outcomes of TNBCs and the expression of DOT1L. Notably, we observed an inverse correlation between DOT1L expression and the RFS rate in patients with TNBC, suggesting that DOT1L may be a therapeutic biomarker of TNBC. Interestingly, F-NepA effectively inhibited the proliferation of human TNBC cells, which constitutively overexpress DOT1L, via the inhibition of both DOT1L activity and the DOT1L-mediated selective suppression of H3K79 dimethylation. Furthermore, a structure–activity relationship study of NepA analogs revealed that the free N^6^-amine group of NepA was necessary for the cytotoxic and inhibitory effects of this agent against DOT1L. We further observed that the introduction of fluorine at the 6′-position of NepA reduced the toxicity associated with the parent compound against a normal breast epithelial cell line. These findings support the possibility that further design and development efforts may yield more bioactive compounds as therapeutic agents against TNBC.

Accumulating evidence suggests that the inhibition of DOT1L-mediated processes may also suppress the metastasis of human cancer cells [56,57]. Despite this possibility, early DOT1L inhibitors, such as EPZ-5676 (developed by Epizyme and Celgene), did not noticeably suppress the growth and metastasis of solid tumors, including BCs [20]. Compared with previously identified DOT1L inhibitors, F-NepA significantly inhibited the migration and invasion of cultured human TNBC cells in vitro. F-NepA treatment also downregulated the protein levels of mesenchymal markers (N-cadherin and Vimentin) while upregulating the level of an epithelial maker (E-cadherin), suggesting that F-NepA can suppress the migration and invasion potential of TNBCs expressing high levels of DOT1L. Moreover, we found that F-NepA is able to effectively regulate the TGF-β-induced EMT processes in both a normal breast epithelial cell and human TNBC cells.

## 5. Conclusion

In summary, our findings highlight DOT1L inhibition as a compelling potential strategy for the targeted therapy of human TNBCs. F-NepA effectively inhibits the DOT1L-mediated methylation of H3K79 and thus suppresses the migration and invasion of TNBC cells. Although an evaluation of the antitumor and antimetastatic activity of F-NepA in animal models is needed, F-NepA appears to be a promising lead compound in the discovery and development of novel therapeutic agents for the treatment of aggressive TNBC.

## 6. Patents

Jeong, L.S. Novel fluoro-homoneplanocin A and nucleoside derivatives, method for the synthesis thereof, and the pharmaceutical compositions for treating cancers containing the same as an active ingredient. KR 20130127883, 2013; US 20130310403, 2013.

## Figures and Tables

**Table 1:** Antiproliferative activities of NepA analogs against a panel of human BC cell lines.

Cell Line	IC_50_ (μM)^a^			
	1	2	3	Etoposide^b^
MCF10A	22.28 ± 1.98	38.97 ± 1.77	>50	31.86 ± 3.45
HCC38	1.22 ± 0.26	0.87 ± 0.17	16.25 ± 2.10	1.36 ± 0.33
HCC1937	0.98 ± 0.14	1.12 ± 0.28	14.36 ± 0.82	6.24 ± 0.39
MDA-MB-231	0.42 ± 0.11	0.47 ± 0.16	9.23 ± 0.69	4.42 ± 0.47
MCF-7	4.94 ± 0.31	5.24 ± 0.09	34.01 ± 1.78	1.11 ± 0.05
T-47D	6.22 ± 0.29	6.17 ± 0.88	29.63 ± 2.33	3.52 ± 0.12

^a^Results are expressed as the calculated half maximal inhibitory concentrations (IC_50_) of test compounds (μM). Data are presented as mean values ± SD (n = 3). ^b^Etoposide was used as a positive control.
